# Prophylactic hyperthermic ıntraperitoneal chemotherapy for radiologic T4 colorectal cancer: impact on locoregional control

**DOI:** 10.1590/1806-9282.20252167

**Published:** 2026-07-31

**Authors:** Vural Argın, Ahmet Orhan Sunar, Nurdan Bezir, Muhammed Ikbal Akın, Ömer Faruk Kandaz, Mustafa Duman, Erdal Polat

**Affiliations:** 1 Marmara University, Pendik Training and Research Hospital, General Surgery Clinic – Istanbul, Türkiye.; 2 University of Health Sciences, Kartal Koşuyolu Yüksek İhtisas Training and Research Hospital, Gastrointestinal Surgery Clinic – Istanbul, Türkiye.

**Keywords:** Colorectal cancer, Peritoneal carcinomatosis, HIPEC, Recurrence

## Abstract

**OBJECTIVE:**

In T4 colorectal cancer, the risk of peritoneal carcinomatosis is high, and the prognosis is poor. The aim of this study was to assess recurrence risk factors, peritoneal recurrence rates, and survival outcomes in patients radiologically diagnosed with T4 tumors who underwent curative surgery with prophylactic hyperthermic intraperitoneal chemotherapy.

**METHODS:**

Between January 2018 and December 2022, 38 radiologically diagnosed T4 colorectal cancer patients who underwent prophylactic hyperthermic intraperitoneal chemotherapy were retrospectively analyzed. All patients had R0 resection; those with intraoperative macroscopic peritoneal or distant metastases were excluded. Demographic data, tumor location, histopathology, and pathological T/N stages were recorded. During follow-up, local/distant recurrence, peritoneal carcinomatosis, metastatic sites, and mortality were assessed.

**RESULTS:**

The median age was 57 years, and 57.9% were male. The primary tumor originated mainly from the colon (81.6%), with moderately differentiated adenocarcinoma being the most common histology (57.8%). Recurrence occurred in four patients (10.5%): 2 hepatic, 1 pulmonary, and 1 peritoneal, with no local recurrence. One patient (2.6%) died within 90 days; no additional mortality was observed. Median overall survival was 55 months (95%CI 49.82–60.90). Univariate analysis showed no significant association between recurrence and tumor location, differentiation, pT or pN stage, or histologic subtype.

**CONCLUSION:**

In cT4 colorectal cancer patients treated with prophylactic hyperthermic intraperitoneal chemotherapy, recurrence was infrequent, peritoneal recurrence was rare, and radiological staging frequently overestimated tumor depth, supporting a potential benefit of prophylactic hyperthermic intraperitoneal chemotherapy in this setting.

## INTRODUCTION

Colorectal cancer (CRC) is a major global malignancy with high morbidity and mortality^1^. Although surgery is the primary treatment for localized disease, recurrence, especially in advanced tumors, remains common^2^. T4 tumors, which invade the serosa or adjacent organs, carry the highest risk of peritoneal metastasis^3^. In these patients, peritoneal recurrence rates reach 17.9% at one year and 36.7% at 3 years after surgery^4^. Once peritoneal carcinomatosis develops, prognosis is poor, with a median survival of only 6–12 months despite systemic therapy^5^. Consequently, recurrence prevention in high-risk T4 patients is clinically essential.

Prophylactic hyperthermic intraperitoneal chemotherapy (HIPEC) has been increasingly used intraoperatively in high-risk patients without visible metastases^6^. The HIPECT4 randomized trial showed a significant reduction in peritoneal recurrence among T4 or mucinous/signet-ring tumors, decreasing rates from 12.4 to 2.4% at 36 months, with an even greater benefit in pathologic T4 disease (1.7 vs. 17.9%; HR 0.09; p=0.003)^7^. In contrast, the COLOPEC (Adjuvant Hyperthermic Intraperitoneal Chemotherapy in Patients With Locally Advanced Colon Cancer) and PROPHYLOCHIP-PRODIGE 15 (Second-look Surgery Plus Hyperthermic Intraperitoneal Chemotherapy Versus Surveillance in Patients at High Risk of Developing Colorectal Peritoneal Metastases) trials did not demonstrate improvements in disease-free survival or prevention of peritoneal recurrence^8,9^. These conflicting results highlight the ongoing uncertainty and the need for further high-quality studies to define the role of prophylactic HIPEC in T4 colorectal cancer^10^.

This study aimed to evaluate risk factors for recurrence and to investigate survival outcomes in colorectal cancer patients radiologically diagnosed with T4 tumors who underwent curative surgery combined with prophylactic HIPEC.

## METHODS

### Study design and patient selection

Between January 2018 and December 2022, 124 patients underwent cytoreductive surgery (CRS) with HIPEC at our institution. Of these, 95 had colorectal cancer, and 29 had pseudomyxoma peritonei. Among the colorectal cancer patients, 57 presented with peritoneal metastases, whereas 38 had radiologically diagnosed T4 tumors and received prophylactic HIPEC without evidence of metastatic disease. The CRS–HIPEC program, initiated in 2018 by two senior surgeons, established a multidisciplinary team, standardized CRS and HIPEC protocols, and implemented perioperative safety measures in accordance with Peritoneal Surface Oncology Group International 2008 recommendations^11^.

### Surgical procedure and hyperthermic intraperitoneal chemotherapy protocol

All surgeries were performed in the Lloyd-Davis position through a midline incision, with previous scars excised to prevent potential tumor implantation. Tumor burden was assessed using the Peritoneal Cancer Index (PCI). CRS was adapted to disease extent and included peritonectomy procedures with organ or bowel resections as required, aiming for optimal cytoreduction (CC-0/1). HIPEC was then administered using a closed technique with mitomycin C or oxaliplatin plus 5-fluorouracil–leucovorin, maintaining 42–43°C for 90 min at a flow rate of 1,000 cc/min through five drains. Intestinal anastomoses were performed after HIPEC, while patients not requiring anastomosis underwent abdominal closure before perfusion.

### Data collection and preoperative assessment

Demographic data (age, sex), primary tumor site, type of surgery, histologic subtype, tumor differentiation, and pathological T and N stages were recorded for all patients. Preoperative staging included thoracic and abdominal computed tomography (CT) to assess resectability and rule out distant metastases. Abdominal MRI was added in cases with suspected liver involvement or mucinous tumor components, while PET-CT was used selectively when further evaluation was required. Serum tumor markers (CEA, CA 19-9, and CA-125) were measured in all patients.

### Postoperative follow-up

After discharge, patients were followed in the medical oncology and gastrointestinal surgery clinics, and recurrences were discussed by a multidisciplinary tumor board. Follow-up included physical examination and serum tumor markers (CEA, CA 19-9, and CA-125) every 3 months for 2 years, every 6 months from years 3 to 5, and annually thereafter. Thoracic and abdominal CT scans were obtained every 6 months for the first 3 years and then annually, or earlier if clinically indicated. Colonoscopy was performed at postoperative years 1, 3, and 5.

### Statistical analysis

Statistical analyses were performed using SPSS version 25.0 (IBM Corp., Armonk, NY, USA). Continuous variables were reported as median (minimum–maximum), and categorical variables were reported as counts and percentages. Associations between recurrence and clinicopathological factors were assessed using the chi-square or Fisher's exact test as appropriate. Overall survival (OS) was analyzed using the Kaplan-Meier method, with group comparisons made using the log-rank test. A p<0.05 was considered statistically significant.

## RESULTS

A total of 38 patients who underwent prophylactic HIPEC were included. The median age was 57 years (range, 24–70), and 22 were male (57.9%). The primary tumor site was the colon in 31 patients (81.6%), the appendix in 6 (15.8%), and the rectum in 1 (2.6%). Surgical procedures included right hemicolectomy in 14 patients (36.8%), anterior resection in 14 (36.8%), left hemicolectomy in 5 (13.2%), low anterior resection in 4 (10.5%), and transverse colectomy in 1 (2.6%). Complete (R0) resection was achieved in all cases. Histopathology showed moderately differentiated adenocarcinoma in 22 patients (57.8%), poorly differentiated in 5 (13.1%), well-differentiated in 3 (7.8%), and mucinous adenocarcinoma in 3 (7.8%). Medullary carcinoma was identified in one patient (2.6%) and low-grade appendiceal mucinous neoplasm in 4 (10.9%). Most tumors were pT3 (52.6%) and pN0 (60.5%); rates of pT4a, pT4b, pN1a, pN1b, and pN2a were 31.6, 10.5, 18.4, 10.5, and 10.5%, respectively (Table 1).

**Table 1:** Baseline clinicopathological characteristics of patients undergoing prophylactic HIPEC.

Variable		n=38 (%)
Age, median (range)		57 (24–70)
Sex		
	Male	22 (57.9)
	Female	16 (42.1)
Primary tumor location		
	Colon	31 (81.6)
	Rectum	1 (2.6)
	Appendix	6 (15.8)
Type of surgery		
	Right hemicolectomy	14 (36.8)
	Transvers colectomy	1 (2.6)
	Left hemicolectomy	5 (13.2)
	Anterior resection	14 (36.8)
	Low anterior resection	4 (10.5)
Type of resection		
	R0	38 (100)
	R1–R2	0
Histological findings		
	Adenocarcinoma well differentation	3 (7.8)
	Adenocarcinoma moderate differentation	22 (57.8)
	Adenocarcinoma poor differentation	5 (13.1)
	Mucinous adenocarcinoma	3 (7.8)
	Medullar carcinoma	1 (2.6)
	Low grade appendiks mucınous neoplasia	4 (10.9)
pT		
	pT1	1 (2.6)
	pT2	1(2.6)
	pT3	20(52.6)
	pT4a	12(31.6)
	pT4b	4(10.5)
pN		
	pN0	23 (60.5)
	pN1a	7 (18.4)
	pN1b	4 (10.5)
	pN2a	4 (10.5)
M		
	M0	38 (100)
	M1	0
Chemotherapeutic agent used in HIPEC		
	Oxaliplatin	36 (94.7)
	Mitomycin-C	2 (5.3)

HIPEC: hyperthermic ıntraperitoneal chemotherapy.

During follow-up, recurrence occurred in four patients (10.5%), including 2 with liver metastases, 1 with pulmonary metastasis, and 1 with peritoneal carcinomatosis. No local recurrence was observed. Within the 90-day postoperative period, major complications (Clavien-Dindo grade III–IV) were recorded in nine patients (23.6%), and mortality occurred in one patient (2.6%). Beyond this patient, no additional mortality was observed during the long-term follow-up. The median overall survival was 55 months (95%CI 49.8–60.9) (Table 2), with a follow-up range of 33–94 months.

**Table 2:** Follow-up.

Variable		n (%)
Development of distant metastases (no PC)		
	Yes	3 (8.1)
	No	34 (91.9)
Site of metastases		
	Liver	2 (66.6)
	Lung	1 (33.4)
Peritoneal carcinomatosis		
	Yes	1 (2.7)
	No	37 (97.3)
Local recurrence		
	Yes	0
	No	38 (100)
	90-day mortality	1 (2.6)
	90-day major complications (Clavien-Dindo III-IV)	9 (23.6)
	Overall survival, month (median)	55 (95% Cl:49.82–60.90)

PC: peritoneal carcinomatosis.

Univariate analysis showed no statistically significant association between recurrence and primary tumor location (p=0.083), histologic subtype (p=1.000), tumor differentiation (p=0.313), pT stage (p=0.089), or pN stage (p=0.158) (Table 3).

**Table 3:** Univariate analysis of risk factors for recurrence in patients undergoing prophylactic HIPEC.

Variable		n (%)	Recurrence (n)	p
Primary tumor location				
	Colon	31 (81.6)	3	0.083
	Rectum	1 (2.6)	1	
	Appendix	6 (15.8)	–	
Histological findings				
	Adenocarcinoma well differentation	3 (7.8)	–	1.000
	Adenocarcinoma moderate differentation	22 (57.8)	2	
	Adenocarcinoma poor differentation	5 (13.1)	2	
	Mucinous adenocarcinoma	3 (7.8)	–	
	Medullar carcinoma	1 (2.6)	–	
	Low grade appendiks mucınous neoplasia	4 (10.9)	–	
Degree of tumor differentiation				
	G1–G2	23 (60.5)	2	0.313
	G3–G4	11 (28.9)	2	
pT				
	pT1	1 (2.6)	–	0.089
	pT2	1(2.6)	1	
	pT3	20(52.6)	1	
	pT4a	12(31.6)	1	
	pT4b	4(10.5)	1	
pN				
	pN0	23 (60.5)	1	0.158
	pN1a	7 (18.4)	1	
	pN1b	4 (10.5)	1	
	pN2a	4 (10.5)	1	

HIPEC: hyperthermic ıntraperitoneal chemotherapy.

## DISCUSSIONS

The findings of our study indicate that prophylactic HIPEC may offer a meaningful oncological benefit by achieving low recurrence rates and favorable survival outcomes in patients with radiologically diagnosed T4 colorectal cancer undergoing curative resection. However, due to the absence of a control group, the observed low rate of peritoneal recurrence cannot be directly attributed to the effect of prophylactic HIPEC and should be interpreted cautiously.

In our study, only one patient (2.7%) developed peritoneal recurrence, and the median overall survival was 55 months (95%CI 33.6–42.4), which is more favorable than previously reported. This low recurrence rate may be partly explained by the fact that 52.6% of patients had pathologically confirmed T3 rather than true T4 tumors, and all surgeries were performed electively under optimal conditions. The HIPECT4 trial reported a 3-year local recurrence rate of 2.4% and demonstrated substantial reductions in regional recurrence with intraoperative HIPEC (absolute risk reduction 10.0%, relative risk reduction 80.6%)^7^. In contrast, the COLOPEC and PROPHYLOCHIP trials did not show a significant benefit of prophylactic HIPEC^8,9^, likely influenced by the predominant use of oxaliplatin and shorter perfusion durations. Although 94.7% of our patients received oxaliplatin, the peritoneal recurrence rate remained comparable to HIPECT4, suggesting that the choice of chemotherapeutic agent alone may not be the determining factor in preventing peritoneal recurrence.

In our study, more than half of the patients classified as T4 on preoperative imaging were ultimately found to have pathologic T3 disease, underscoring the limitations of CT in accurately assessing serosal invasion^12^. This substantial discordance indicates a high rate of radiologic overstaging and may partly explain the favorable oncologic outcomes observed in our cohort. Peritumoral inflammation, fibrosis, or desmoplastic reaction may mimic serosal involvement and lead to overstaging, as can close proximity to adjacent organs^13,14^. Similar discrepancies were noted in the HIPECT4 trial, where 29.2% of radiologic T4 cases were downstaged to T3 on pathology^7^, and Shkurti et al. reported only 59% concordance between radiologic and pathologic T stage, with 53% agreement specifically for T4 tumors^15^. These findings suggest that relying solely on radiological staging may risk overtreatment when selecting patients for prophylactic HIPEC. Therefore, radiologic T4 classification alone should be interpreted cautiously and ideally integrated with intraoperative assessment and additional high-risk features when considering prophylactic HIPEC indications. Accordingly, the high proportion of pathologic T3 cases in our cohort likely contributed to the recurrence outcomes observed.

In our study, early postoperative outcomes were acceptable, with a 2.6% mortality rate and a 23.6% major morbidity rate (Clavien-Dindo III–IV). These results align with published data reporting mortality of 1–5% and major morbidity of 20–35% after CRS–HIPEC^16^, indicating that prophylactic HIPEC can be safely performed in experienced centers with proper patient selection. The median overall survival in our cohort was 55 months, which compares favorably with similar trials such as PRODIGE 7 (Cytoreductive Surgery Plus Hyperthermic Intraperitoneal Chemotherapy Versus Cytoreductive Surgery Alone for Colorectal Peritoneal Metastases) (41.7 months)^17^ and PROPHYLOCHIP (51 months)^9^. This extended survival likely reflects the high proportion of pathologic T3 cases (52.6%) in our series. Nonetheless, when combined with the low rate of peritoneal recurrence, these findings suggest a possible benefit rather than establishing a definitive oncologic advantage of prophylactic HIPEC in appropriately selected high-risk colorectal cancer patients.

In our analysis, none of the clinicopathological variables showed a statistically significant association with recurrence, likely due to the limited sample size and low number of events. The study; therefore, had limited statistical power, and multivariable analysis could not be reliably performed for this reason. Although T4 stage, poor differentiation, and colon localization are recognized risk factors for peritoneal recurrence in the literature^18,19^, these associations were not detectable in our cohort. The universal application of prophylactic HIPEC may also have reduced the observable impact of individual risk factors. Overall, the trends remain consistent with published data, but larger studies are needed to validate these findings.

### Limitations

This study has several limitations. Its retrospective, single-center design introduces potential selection and data collection bias. The relatively small sample size (n=38) limits statistical power, especially for subgroup analyses. In addition, the short follow-up for some patients may not fully capture long-term survival or late recurrences. Variability in HIPEC protocols, particularly the use of different chemotherapeutic agents, adds treatment heterogeneity. Lastly, the absence of a control group prevents a definitive assessment of the true impact of prophylactic HIPEC on survival and recurrence outcomes.

## CONCLUSION

In patients with T4 stage colorectal cancer who underwent prophylactic HIPEC, the overall recurrence rate was 10.5% (4 out of 38 patients). Neither T stage, N stage, nor tumor differentiation showed a statistically significant association with recurrence; however, this may be attributable to the limited sample size. Importantly, the peritoneal recurrence rate was low, and more than half of the patients initially staged as radiologic T4 (52.6%) were subsequently found to be pathologic T3. These findings underscore both the potential role of prophylactic HIPEC in reducing peritoneal recurrence and the limitations of radiologic staging. Larger prospective randomized trials are warranted to validate these results.

## Data Availability

The datasets generated and/or analyzed during the current study are available from the corresponding author upon reasonable request.
